# Monitoring and Root Cause Analysis of Clinical Biochemistry Turnaround Time at a Tertiary Care Institute

**DOI:** 10.7759/cureus.39821

**Published:** 2023-06-01

**Authors:** Priyanka Prasad, Rakesh Kumar, Santosh Kumar, Poonam Sinha

**Affiliations:** 1 Department of Biochemistry, Nalanda Medical College and Hospital, Patna, IND; 2 Department of Pediatrics, Nalanda Medical College and Hospital, Patna, IND; 3 Department of Biochemistry, Indira Gandhi Institute of Medical Sciences, Patna, IND

**Keywords:** root cause analysis, clinical biochemistry, laboratory, samples, turnaround time

## Abstract

Introduction: Most laboratories around the world have focused on improving the analytical quality of laboratory tests. Laboratory turnaround time (TAT) is often left unnoticed and under-recognised in the healthcare setting. Both patients and clinicians are more interested in receiving rapid, reliable, and accurate results. This can be achieved by improving the TAT through the identification of the causes that lead to delayed TAT.

Materials and methods: This prospective study aims to identify the cause of delayed TATs within the outpatient department and implement corrective strategies to overcome them. A total of 214 samples were received. The study was conducted for a period of two years; of all the samples received, 154 were from the outpatient department, and 78 samples exceeded the expected TAT. The samples were analysed in the clinical biochemistry department of the hospital. The time spent at each station was determined using an internal computer system, which was also used to identify the samples that exceeded TATs. The primary outcome of the study was to identify the number of samples exceeding TAT and the causes of it.

Results: Upon implementation of corrective measures and root cause analysis, the TATs were reduced from 80-88% to 11-33%. After analysing the duration of time for the samples that exceeded TAT, 45.1% and 37.5% exceeded 30 minutes in Year 1 and Year 2, respectively. Only 3.2% and 6.2% exceeded five hours in Year 1 and Year 2, respectively. Furthermore, using root cause analysis, it was found that 12% of the delay was due to increased waiting time or sample collection, 14% included other causes such as outsourcing of samples, and 18% of the delay was due to pre-analytic processing time.

Conclusion: Our study concludes that TAT is an important quality assessment tool within the laboratory setting, and with proper identification of causes, it can be improved. Although monitoring TAT is a tedious process that mandates tremendous efforts, with the presence of real-time monitoring, improving TAT is an achievable goal. This, in turn, can improve patient treatment outcomes and clinician satisfaction.

## Introduction

Delays in laboratory response have a significant impact on patient outcomes. Laboratory services are built on four pillars: accuracy, timeliness, authenticity, and precision. All four of these factors are critical in the care and treatment of patients. However, timeliness is an equally crucial factor in determining how quickly a patient receives the correct treatment [1]. Despite the efforts of laboratory professionals to incorporate multiple models to improve timeliness, it remains a challenge [2]. Furthermore, studies have shown that practitioners favour rapid point-of-care testing due to its capacity to provide quick results and the ease of sampling near the patient [3].

While analysing various time intervals and their effect on service, it is important to be aware of the various definitions in use. One of the most commonly used terms in laboratory services is turnaround time (TAT) which indicates the time between sample collection and delivery of verified results [4]. However, there are varied definitions of TAT in our study. Why it is then laboratory professionals have difficulty avoiding delays in reporting laboratory results? The answer is reducing the TAT is a difficult task. It mandates identifying the root causes that result in such delays. TAT is also regarded as one of the most noticeable features by both clinicians and patients in determining the quality of laboratory service given [5].

Root cause analysis is an analytical tool used by hospitals to improve the quality of care for patients and has been in use in the healthcare sector for two decades [6]. In order to improve performance over the long run, this study used root cause analysis to pinpoint the most likely causes of issues, complaints, and undesirable events that resulted in a delay in TATs. The goals of this study were to assess the TAT of frequently requested laboratory tests at the laboratory of this tertiary care facility, identify the causes of longer TATs, and then explore possible solutions.

## Materials and methods

This is a prospective study carried out at the Department of Biochemistry, Indira Gandhi Institute of Medical Sciences, Patna. A total of 214 samples were received, and the study was carried out over a two-year period. We made the decision to rate the hospital's services according to their quality, and TAT was one of them. In our study, TAT was defined as the time taken from the point of sample collection until the lab results are dispatched to the patient [7]. There have been disputes about the accurate definition of laboratory TAT; nevertheless, we have tried to incorporate the most ideal definition for our study setting.

A consensus was developed between the Head of the Department of Biochemistry, Indira Gandhi Institute of Medical Sciences, Patna, the Laboratory Director, and the clinicians to identify the TAT for various laboratory test results as it is enlisted in Table 1.

**Table 1:** Laboratory TATs ALT: alanine transaminase, AST: aspartate transaminase, ALP: alkaline phosphatase, LDH: lactate dehydrogenase, TC: total cholesterol, HCL: high-density lipoprotein cholesterol, TSH: thyroid stimulating hormone, PSA: prostate-specific antigen, TIBC: total iron binding capacity, HbA1c: glycated hemoglobin, G6PD: glucose-6-phosphate dehydrogenase, ADA: adenosine deaminase, LFT: liver function tests, TFT: thyroid function tests, FSH: follicular stimulating hormone, LH: luteinising hormone, HCG: human chorionic gonadotropin

Parameters	TAT (in hours)
Glucose, creatinine, urea, uric acid, total protein, albumin, ALT, AST, bilirubin, ALP, calcium, magnesium, phosphorus, LDH, TC, HDL-c, amylase, lipase	1.5
Total PSA, TSH, ferritin, iron, TIBC, HbA1c	2
G6PD, ADA, LFT, TFT, lipid profile, serum osmolality, urine osmolality, vitamin B12, FSH, LH, prolactin, HCG	3

**Figure 1:**
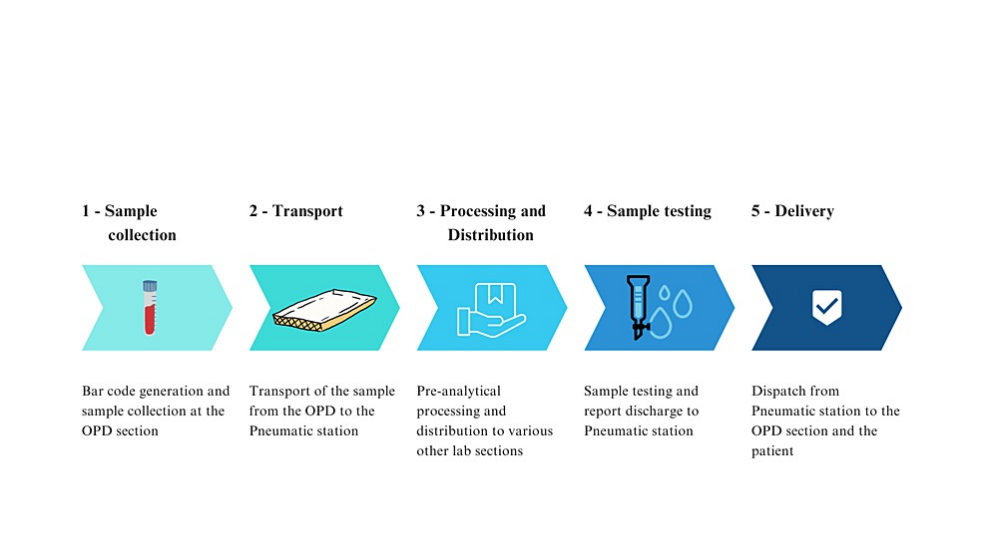
Various stations from sample collection to delivery to the patient

The time taken from the outpatient department (OPD) sample collection to the delivery of laboratory results to the patients has been documented through five steps, represented in Figure 1 below.

Therefore, the figure illustrates that there are three phases: (1) pre-analytical, (2), analytical, and (3) post-analytical.

An internal computer system was employed to both identify the samples that exceed TATs and to calculate the amount of time spent at each station. The reasons for the delay of samples at each station were recognised and noted by laboratory professionals as well as resident physicians. During the monthly sessions, the data collected were assembled, examined, and discussed in order to determine potential preventative measures. The study was approved by the institutional ethics review board (Indira Gandhi Institute of Medical Sciences Institutional Ethics Committee; Letter Number: 623/IEC/IGIMS/2018).

## Results

The study was conducted for a period of two years. Around 214 samples were sent to the study site's Clinical Biochemistry department for evaluation during this time period, and 154 of those samples came from the OPD. Furthermore, 78 samples exceeded the desired TAT, according to our data.

**Figure 2:**
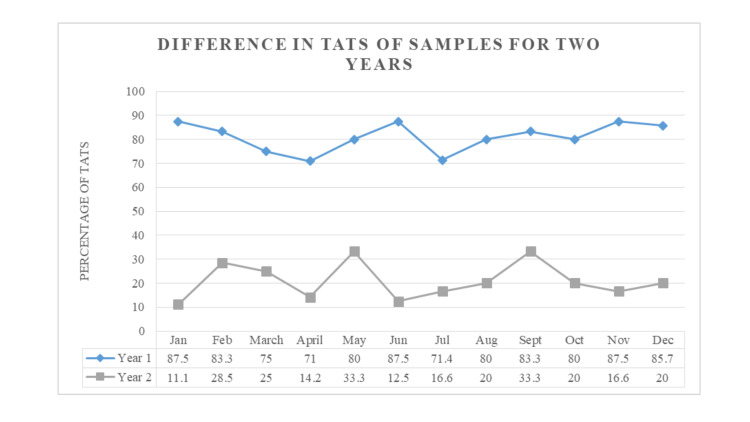
Comparison of the samples with increased TATs in the two years

Table 2 and Figure 2 given below depict the number of samples received and a comparison of the change in TAT for the two years studied.

**Table 2:** Number and percentage of samples that exceeded the TAT OPD: outpatient department, TAT: turnaround time

Year/month	Parameters	Jan	Feb	Mar	Apr	May	June	Jul	Aug	Sep	Oct	Nov	Dec
Year 1	Total no of samples	10	9	6	10	9	10	11	6	10	11	10	8
	Total no of OPD samples	8	6	4	7	5	8	7	5	6	5	8	7
	Total no of samples exceeding the TAT	7	5	3	5	4	7	5	4	5	4	7	6
	% of specimens exceeding TAT	87.5	83.3	75	71	80	87.5	71.4	80	83.3	80	87.5	85.7
Year 2	Total no of samples	10	9	9	8	8	9	9	7	10	9	8	8
	Total no of OPD samples	9	7	8	7	6	8	6	5	6	5	6	5
	Total no of samples exceeding the TAT	1	2	2	1	2	1	1	1	2	1	1	1
	% of specimens exceeding TAT	11.1	28.5	25	14.2	33.3	12.5	16.6	20	33.3	20	16.6	20

As shown in Table 1, despite an increase in workload for the second year, we were able to shorten the TAT from 80-88% to 11-33% (ranging from 12 months in Years 1 and 2).

We examined the stages in which the delay occurred and determined their causes by dividing the TATs across the timeframe for each month. According to the observations, TATs over five hours were primarily caused by the outsourcing of the samples, the delivery of results the following day due to late sample collection (after OPD visits), significant technical problems (breakdown of the analysing equipment), or by incorrectly entering the time into the system. TATs of two to five hours were primarily brought on by sample or misplaced test findings. Finally, the TAT ranging from 30 minutes to two hours was due to the time needed for various process-related factors (such as dilution), clinical correlation, and tests that needed additional corrective actions (Table 3).

**Table 3:** Number of samples exceeding TAT classified as per the time duration OPD: outpatient department, TAT: turnaround time

Time taken	Year 1	Year 2	Total
<30 minutes	28 (45.1%)	6 (37.5%)	34 (43.5%)
30 minutes-1 hour	8 (12.9%)	4 (25%)	12 (15.3%)
1-2 hours	13 (20.9%)	2 (12.5%)	15 (19.2%)
2-5 hours	11 (17.7%)	3 (18.7%)	14 (17.9%)
>5 hours	2 (3.2%)	1 (6.2%)	3 (3.8%)
Number of samples			
OPD samples exceeding TAT	62 (81.5%)	16 (20.5 %)	78 (50.6 %)
Total OPD samples received	76	78	154
Total number of samples received	110	104	214

Corrective actions were implemented to avoid TAT delays during the pre-analytical, analytical, and post-analytical stages. The monthly meetings, which were overseen by a biochemist consultant, were where the corrective steps were discussed and were intended to be implemented (Table 4).

**Table 4:** Root cause analysis with possible corrective measures OPD: outpatient department, TAT: turnaround time

Phases	Causes of delay	% contribution to delayed TATs	Corrective measures
Pre-analytical	Incorrect entry of patient details due to illegible handwriting	<1	Implement a computerised physician-ordered bar code entry system
	Tests requiring repeated sampling and/ or hemolysis	<1	Training phlebotomists and using advanced techniques (than syringes and needles)
	Waiting time and sample collection delay	12	Proper management of sample collection without compromising the quality
	Time is taken for pre-analytical processing (centrifugation and distribution)	18	Employing more efficient personnel and also using more than one processing instruments
	Time is taken to receive samples from other labs	1	Distribute the samples at the time it is received at the Pneumatic station
Analytical	Delay in testing due to increased sampling time or technical glitches	4	Proper maintenance of testing equipment, regular cleaning and updation, and proper human utilisation
	Misplaced samples during delivery	<1	Ensuring that the one receiving the sample is assigned with the processing of it
	Need to re-run the test and also for sample dilution	2.5	Placing auto-run dilution to reduce time lags
Post-analytical	Delay in reading the results from the test equipment to the laboratory information system	5	Linking all the laboratory equipment with the laboratory information system
	Validation of the test results obtained	1	Timely review by clinicians and confirmation of the diagnosis through the laboratory information system
	Test results are misplaced either at the pneumatic station or in various labs	3	Ensuring proper training and education for the staff
	Test results dispatched after 6 pm or after the OPD closures	<1	Engaging in active alert to the treating clinician, if an emergency case is detected
All phases	No specific reason	36	A delay of 5 to 10 minutes is acceptable and TATs of less than 30 minutes should be accepted
	Other causes include outsourcing samples for testing	14	Redefining TATs for such inevitable scenarios with a different process to calculate TAT

## Discussion

Opportunities to improve the quality of laboratory medicine include improvements in the pre-analytical, analytical, and post-analytical phases [8]. Any stage of the testing cycle, from sample collection to the interpretation of the test results, is susceptible to laboratory errors. The analytical stage, which falls under the laboratory's purview, has traditionally been the focus of quality programmes. These programmes are crucial for accurate and dependable results, but quality improvement programmes in the pre- and post-analytical programmes can be a bigger source of quality improvement [9-11]. In the post-analytical phase, the most common cause of negative outcomes are increased TATs, delay in delivery of reports or delay in the interpretation of test results [12]. Accurate results if received late or in the wrong hands can interfere with quality outcomes [13]. Therefore, the healthcare system needs to identify the root reasons for elevated TATs and put improvement plans into place. Prior to adopting the root cause analysis, the TATs in our study were found to be between 80% and 88%, and after doing so over the course of two years, they decreased to about 11% to 33%.

The majority of studies carried out over the years have concentrated on determining TATs based on the time spent on each test. To give a better understanding of the current laboratory practice, this study has determined pre-defined values and also the number of tests that exceed the cut-off value. The level of specificity of the laboratory test ordered, the deadline for receiving test results, and the type of patient for whom the test is ordered all influence estimates for the ideal TAT. Clinicians and laboratory scientists have long disagreed over the appropriate TAT for various prescribed tests. [14]. Therefore, a consensus has to be reached based on the local demographics as done in our study to ensure positive treatment outcomes.

Common delays for the samples with increased TATs were identified in our study. Cakirca et al. [15] have identified that insufficient volume is the most common cause of sample rejection in the hematology laboratory and the second leading cause in the biochemistry laboratory. Furthermore, Chawla et al. [16] have also found that the number of samples with insufficient volume was higher in the OPD than in the inpatient samples. However, other factors found in our study that contributed to an increase in pre-analytical TAT included longer waiting times (12%) and pre-analytical processing times (18%). The authors hypothesised that the local workload and the prevalence of incorrect phlebotomy procedures were responsible for this variation in the observed delays for TATs.

In order to improve patient outcomes, the laboratory TAT must be decreased. Kaur et al. [17] have reported that delay in sample transportation is one of the many causes for delayed pre-analytical TAT in the OPD department and was found to be 50.4±11.9 minutes. Furthermore, poor phlebotomy techniques also result in a longer pre-analytical TAT [18]. Nevertheless, 14% of the longer TATs are attributable to the outsourcing of samples. This may be due to a lack of transportation employees or a hospital staff that is unaware of the consequences of delayed transportation on patient outcomes However, Fernandes et al. [19] have suggested that the presence of a pneumatic tube system can shorten the transport time of samples. The hospital laboratory under evaluation in our study has a pneumatic station in place and investigates the other factors that may have contributed to the delayed TATs.

Furthermore, Bilwani et al. [20] have concluded that 40% of the delays in TATs were primarily due to machine breakdown, 36% due to delays in the maintenance of the laboratory equipment, and 18% due to staff negligence. Similar results were seen in our investigation, where 4% were attributed to technical issues and 1% to misplaced samples. In our investigation, non-analytical delays accounted for 50% of the TAT delays, and 14% of those delays were attributable to outsourcing of the samples. In order to deploy updated techniques and enhance patient outcomes, there is a requirement for periodic monitoring of delayed TATs. Coetzee et al. [21] have recommended that weekly monitoring of TATs identifies poor performance more accurately than aggregate reporting. Future laboratory personnel needs to implement weekly analysis, thereby focusing on the identification of poorly performing laboratories that need immediate intervention.

Although this study is unique, there were a few limitations. The TATs presented in this study only include those that were entered into a laboratory information system, as there was no end-to-end monitoring system used for TAT monitoring. Furthermore, the sample size is small, and future studies with larger sample sizes will ensure the generalisability of the study results. The study has engaged in aggregate reporting; however, frequent reporting could have enabled active retrieval of causes and implementation of updated interventions. The study focuses especially on the OPD, and subsequent research must pinpoint specific areas that need improvement across different laboratories.

## Conclusions

Overall, our investigation into TAT evaluation shows that there are numerous reasons why TATs are delayed and that they can be improved with cooperation from multiple departments. The timeliness of laboratory findings can be considerably improved with prompt interventions targeted at causes of delays. Although manually monitoring TATs is a laborious procedure, the development of artificial intelligence and technology has overcome these technological challenges, allowing for real-time monitoring of TATs. Hence, real-time capturing with proactive interventions can improve patient satisfaction.
